# Nutritional and lifestyle management of the aging journey: A narrative review

**DOI:** 10.3389/fnut.2022.1087505

**Published:** 2023-01-24

**Authors:** Amira Kassis, Marie-Claire Fichot, Marie-Noelle Horcajada, Astrid M. H. Horstman, Peter Duncan, Gabriela Bergonzelli, Nicolas Preitner, Diane Zimmermann, Nabil Bosco, Karine Vidal, Laurence Donato-Capel

**Affiliations:** ^1^Whiteboard Nutrition Science, Beaconsfield, QC, Canada; ^2^Nestlé Research, Société des Produits Nestlé S.A., Lausanne, Switzerland

**Keywords:** aging, physiological changes, metabolism, nutrition, lifestyle

## Abstract

With age, the physiological responses to occasional or regular stressors from a broad range of functions tend to change and adjust at a different pace and restoring these functions in the normal healthy range becomes increasingly challenging. Even if this natural decline is somehow unavoidable, opportunities exist to slow down and attenuate the impact of advancing age on major physiological processes which, when weakened, constitute the hallmarks of aging. This narrative review revisits the current knowledge related to the aging process and its impact on key metabolic functions including immune, digestive, nervous, musculoskeletal, and cardiovascular functions; and revisits insights into the important biological targets that could inspire effective strategies to promote healthy aging.

## 1. Introduction

The average age of the global population is increasing rapidly, driven by multiple factors such as longer life expectancy and reduced birth rates in both developed and developing countries (1). According to the 2019 United Nations report on aging (1), the number of people 65 years and older will have doubled globally by 2050, and it is estimated that a quarter of our lifetime will be spent after 65 years. With a longer lifespan comes increasing chronic disease risk for the individual and burden for the health care system. Health economic studies show that despite increasing longevity, the number of years lived in poor health have increased in the last 30 years (2). More specifically, non-communicable diseases have become the largest cause of Disability Adjusted Life Years (DALYs) (3), and are most prevalent in the population above 40 years with a prevalence estimate of 65–98% of multimorbidity in individuals over 65 years of age (4). Furthermore, chronic conditions in older adults are tightly linked to quality of life and social functioning (5, 6).

A sustained high quality of life should be a priority for aging societies; however, health and wellness are often compromised in the reality of older adults, and exacerbated by poor nutrition, low physical activity and poor sleep quality (7, 8). Therefore, in designing nutrition and lifestyle solutions that promote health span and quality of life in aging populations is warranted.

**FIGURE 1:**
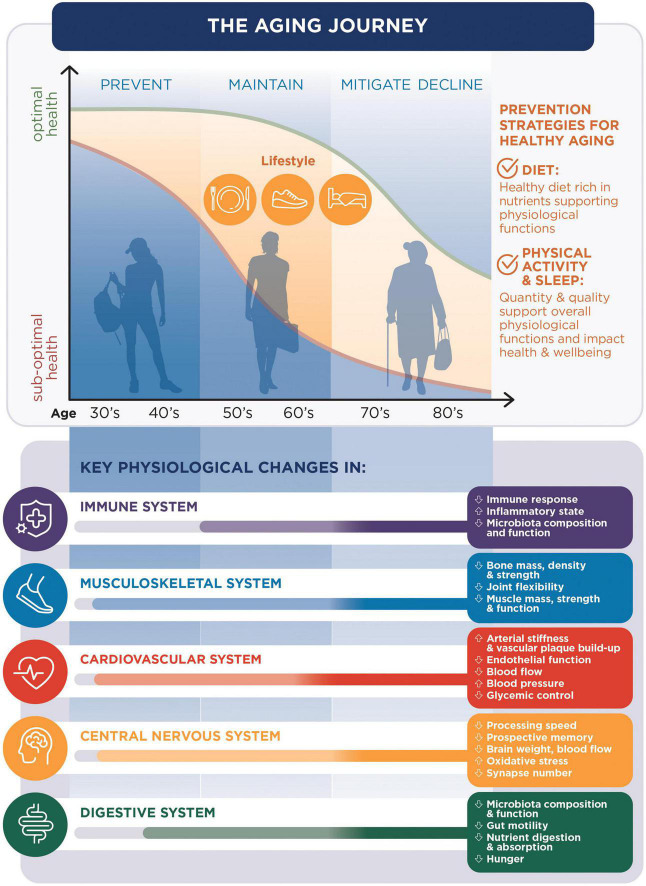
The aging journey: The changes in health conditions with aging [being optimal (green line) or suboptimal (orange line)] are related to changes in key physiological functions and can be prevented with diet, sleep and physical activity.

Developing such solutions should start with a good understanding of the aging process and its impact on the different aspects of health. In this comprehensive review, we describe aging as a multisystem process. We also identify physiological changes in the aging body that should be used as targets to develop dietary and lifestyle solutions for the maintenance of good health in older individuals (Figure 1). In this review, we present a selection of the most promising nutritional and lifestyle solutions shown to be associated with the maintenance of good health in aging.

## 2. What is aging?

Why and how we age have long been the questions driving numerous scientists to develop multiple theories about aging. Whether aging is driven more by genetics or the environment, it is the product of both, accounting for the variability in biological age amongst individuals of a similar chronological age. Busse et al. first described aging in 1969 as being two-fold: the cellular changes in structure and function that affects organs and systems, termed “primary aging,” and the changes caused by the interaction of primary aging with the environment, diet, lifestyle, and diseases, termed “secondary aging” (9). More recently, in their 2019 report on aging, the WHO defines the “primary” path or trajectory of aging as “intrinsic capacity,” while “functional ability” represents the aging trajectory aided by the environment (10).

To answer the question of why we age, the concept of hallmarks of aging was first put forth by Lopez-Otin in 2013 (11) and later revisited by Andrew Steele in his book “Ageless” (12), and David Sinclair in his book “Lifespan” (13). Briefly, these hallmarks are biological changes associated with aging in a way that their evolution determines the rate of aging. The nine hallmarks identified by Lopez-Otin et al. are (1) genomic instability, (2) telomere attrition, (3) epigenetic alterations, (4) loss of proteostasis, (5) deregulated nutrient-sensing, (6) mitochondrial dysfunction, (7) cellular senescence, (8) stem cell exhaustion, and (9) altered intercellular communication; a list to which Steele adds the tenth hallmark: changes in the microbiome. Based on recent advances in the field of aging, modulating these hallmarks through diet and lifestyle is key to delaying the path of age-related decline.

## 3. Physiological and functional changes in the aging adult

The hallmarks of aging are characteristic of every aging cell in our body, the result being changes at the level of the different organs and systems. In the section below, we discuss these changes affecting the main biological systems in the body, namely the digestive, immune, musculoskeletal, nervous, and cardiovascular systems, as well as the microbiome.

### 3.1. The digestive system

The role of the digestive system goes beyond nutrient supply and includes hormone production, immune regulation and gut-organ communication, important functions impacting the main biological systems in the human body (14). The gastrointestinal (GI) tract is home to billions of important residents, the gut microbiota, known for their role in gut barrier protection, nutrient digestion and absorption (15), and communication with other organs (16). Age-related changes can be observed along the entire digestive tract, some starting as early as age 40 in a healthy population (17). Changes in food intake control that impact hunger and satiety, as well as altered oral function (18), gut integrity (19), motility, digestion, and absorption may be observed around the 4th decade of life and are highly prevalent by the age of 75. Gastro-esophageal reflux, a condition associated with decreased esophageal function, is more prevalent in older versus younger men and women (20). Similarly, motility at the level of the colon is compromised with age, as suggested by studies looking at rates of constipation in young versus older adults (14). Although these studies are often confounded by secondary aging factors such as physical activity, dietary habits, and water intake, reduced colonic motility is an age-related issue and a common complaint reported to affect quality of life (21, 22). Food intake control is another area that is believed to be affected by aging. More specifically, lower hunger ratings and higher level of anorexigenic hormones [namely cholecystokinin (CCK)] in the elderly have been reported versus their younger counterparts (14) and further supported reduced energy intakes in older adults (23). However, significant changes are likely to occur closer to the age of 70, an age at which the effect of these alterations leads to anorexia and involuntary weight loss in the elderly (24). Meanwhile, the absorption of some important micronutrients such as calcium, vitamin D and vitamin B12 declines with age (25). Vitamin B12 absorption is dependent on pepsin and acid secretions shown to be lower in older adults (14). Calcium absorption is mediated by the intestinal response to the active form of vitamin D which is impaired with age. This is compounded by the age-related reduction in intestinal and renal absorption of vitamin D as well as its synthesis in the skin (26). On the other hand, and although lactose intolerance is a common complaint of older adults, research to date has not confirmed a decrease in lactase levels between young and old age (27). Nevertheless, perceived, or self-diagnosed lactose intolerance may lead to a limited consumption of dairy products and therefore lower calcium intake.

Finally, and while the impact of host aging on microbiota function has not been clearly determined, the shift in bacterial composition (discussed in the section “3.3. The gut microbiome”) implies a risk of pro-inflammatory state in the gut, associated with digestive and absorptive disturbances (28).

### 3.2. The immune system

The immune system is developed throughout infancy until adulthood (29). Maintaining a healthy immune system leading up to the onset of aging and throughout this life stage is an investment into the prevention of infections and diseases of old age. To tackle age-related immune decline, nutritional and lifestyle solutions should be targeting its three main components: immunosenescence, inflammaging, and dysbiosis. Immunoscenescence is a reduction in quality and quantity of immune responses, resulting from an imbalance in the type of immune cells, their ability to mount an adequate immune response against pathogens and to build memory of previously encountered pathogens (30, 31). It is also paradoxically characterized by an excessive inflammatory response to antigens and an ineffective resolution of inflammation, favoring a pro-inflammatory state, which when chronic, is referred to as inflammaging (31, 32). Although multifactorial, inflammaging is mainly driven by three aspects: a dysregulation of the innate monocyte-macrophage network (innate immunity), a gradual senescence of T and B cells (adaptive immunity), and external amplifying factors such as the lifelong exposure to antigens and inflammatory stimuli (31, 33, 34). Consequences of immune cell senescence, such as the release of mitochondrial DNA into the plasma, are observed as early as age 50, stimulating the production of pro-inflammatory mediators typical of inflammaging (31, 35). Reduced vaccine responsiveness and increased risk of chronic diseases are examples of age-related features associated with inflammaging (33–35). Finally, the immune system is the orchestrator of the collaboration between gut microbiota and its host, also acting as a surveillance system to ensure that microbial balance remains in favor of commensal bacteria over pathogens. Immunosenescence therefore greatly affects this balance, leading to dysbiosis. In return, dysbiosis affects the production of anti-inflammatory cytokines, vitamins, and immune cells, exacerbating low-grade inflammation and the aging process in the gut. This reduces gut integrity and increases susceptibility to pathogenic infections. The main health consequences of age-related immune decline are a higher susceptibility to newly encountered pathogenic infections and a potential reduction in vaccination responses in older adults.

### 3.3. The gut microbiome

Although not an organ system *per se*, the gut microbiome plays a pivotal role in the health of other systems, be it immune, digestive, cardiovascular, musculoskeletal, or nervous systems (36). A healthy microbiota, generally characterized by high counts of bifidobacterium and lactobacillus species, compete with pathogens for adhesion to the intestinal mucosa and promote the development of immune cells. In addition, the production of short chain fatty acids (SCFA), a major asset of healthy microbiota, reduces the luminal pH making it a hostile environment for pathogens (37, 38). That said, the microbiome has a well-recognized impact well beyond the gut, on overall health. Indeed, it is increasingly clear that an immune dysfunction at the level of the gut may have consequences on other organ immunities (30). This is mediated by the crosstalk between different systems through the so-called gut-organ axes, such as the gut-liver axis, gut-lung axis and the gut-brain axis (16).

Just like other systems, the microbiome is prone to aging. Studies having examined changes in microbiota composition and diversity across life stages agree that major shifts in microbiota composition occur with the onset of frailty (39). While microbiota during adulthood is fairly stable, it is not clear what age is associated with the decline in microbial richness and diversity (39). However, studies comparing young versus old adults generally show that “young microbiota” profiles tend to be enriched by taxa such as Clostridiales and Bifidobacterium, while “old microbiota” profiles are generally enriched in Proteobacteria and pathobionts (31, 40). This microbial imbalance is associated with a high level of pro-inflammatory cytokines and low levels of SCFA, disrupting the stability of intestinal epithelial tight junctions (41). The resulting increase in gut permeability allows pathogens to translocate into the systemic circulation.

### 3.4. The central nervous system

Brain aging is believed to start in the late 20s with brain shrinkage of about 5% per decade after age 40 (42, 43). Data from the Centers for Disease Control and Prevention (44) surveys show that one in nine adults over 45 years of age self-report subjective cognitive decline (45). Behind this decline lies a series of structural changes (46) such as brain weight, neuron numbers and size of dendritic fields (47), as well as neurophysiological changes such as cerebral blood flow, myelination and synapse numbers to name a few (48–51). These changes can be linear (cerebral blood flow and glucose metabolism) or follow a bell- shaped curve with a decline starting as early as 45 years of age (e.g., myelination), yet others have more complex trajectories across life stages (e.g., synapse numbers). The latter is considered to be the most strongly correlated with cognitive impairment (52, 53).

Age-related changes in the brain were traditionally believed to affect all brain regions equally; however, it is increasingly clear that these changes are region-specific (54, 55). The most affected areas are the frontal cortex and parts of the hippocampal system, regions involved in executive function, learning and memory (47, 56, 57). Indeed, the age-associated loss of gray matter (consisting of neuronal cell bodies) with age is especially evident in the lateral prefrontal cortex, hippocampus, cerebellum, and caudate nucleus; and the shrinkage of white matter (consisting of myelinated axons) is seen to be particularly prevalent in the prefrontal cortex (54).

Changes in neurotransmitter levels have also been observed with aging, namely dopaminergic and cholinergic declines, potentially compromising attention and memory (49, 54). Overall, and from a cognitive function point of view, age-related deficits have been observed at the level of the three main cognitive domains: attention, memory, and executive function. However, within these domains, not all functions are equally affected. For instance, in the attention domain, processing speed clearly declines with age (58, 59) while sustained attention was surprisingly seen to improve in older adults as subjects trade off reaction time for increased accuracy in response (59). This was supported by a meta-analysis by Vallesi et al., who reported a consistently longer reaction time in older adults contrasted with a higher accuracy of response on sustained attention tasks in the older versus younger adults (59). Similarly, in the memory domain, spatial memory which pertains to remembering the location of objects, and episodic memory used to recall past events are seen to decline by 20–40% between the ages of 60 and 78 years (60). On the other hand, implicit memory and semantic memory seem fairly stable in healthy aging (60, 61). As for the domain of executive functions, its 3 core abilities are not equally affected by age. Indeed, while working memory has been reported to decline starting at age 20, inhibition is not necessarily affected by age and if so, the decline is task specific. This is particularly observed in cases of task switching, affected mostly in dual task contexts (62). Along with cognition, mood and well-being are important players in maintaining brain health as we age. Interestingly, well-being appears to generally be stable or even improve with age, when physical health and cognitive impairment are ruled out as potential confounders (63–68). Despite the fact that this life stage is often characterized by the loss of loved ones, retirement and financial insecurity, older adults choose to focus on positive thoughts and to disregard negative memories or stimuli, seeking direct gratification rather than long-term reward and therefore maintaining a more positive outlook on life than younger adults (67). This phenomenon was coined by Reed et al. as “the age-related positivity effect” (67, 69).

### 3.5. The musculoskeletal system

Musculoskeletal health is crucial to support a level of mobility that humans require to be physically independent and autonomous. It is therefore a very strong predictor of quality of life (70, 71). Osteoporosis, osteoarthritis, sarcopenia and cachexia are conditions affecting the aging adult with a heavy impact on mobility and consequently, quality of life (72).

Osteoporosis is defined as a “skeletal disorder characterized by compromised bone strength predisposing a person to an increased risk of fracture” (73). It is estimated that over 200 million individuals globally and 30% of women in Europe and the US (74) suffer from osteoporosis. Age-related bone mass loss is estimated to be around 1–2% per year and bone strength loss around 1.5–3% per year in individuals over 50 (75), an age beyond which bone mineral density (BMD) is strongly correlated to the risk of fracture. Although the general trend is a declining one, there are important gender differences at every life stage resulting in peak bone mass being lower and its decline higher in females compared to males, with an accelerated rate observed in women around menopause (75). In addition, after age 50, the risk of having an osteoporotic fracture is 53% for women and 21% for men, agreeing with the trend observed for BMD (76).

Osteoarthritis (OA) is the most common joint disease affecting about 37% of adults 60 years and older in the US and causing a major loss of mobility and independence in older adults over 65 (77). OA affects the weight-bearing joints and is characterized by a degradation of the cartilage matrix leading to symptoms such as chronic pain, joint stiffness, and instability (77). Numerous cohort studies have investigated the prevalence of OA using the radiographic presence of OA in the knee, hip and other joints (78). Data show a prevalence of knee OA close to 40% in individuals over 60 with a 10% increase between ages segment of 60–70 and 80 and beyond. Hip OA similarly increases by 15% between ages 40 and 85 years and over (78).

On a cellular level, the progression of OA occurs through a vicious circle whereby cartilage degradation stimulates chondrocyte proliferation and associated catabolic factors, further degrading the cartilage tissue (77). Normal age-related wear and tear can lead to a progressive degradation of the cartilage. Additional factors such as obesity, loss of body balance, joint injury and instability can accelerate the process of cartilage loss with its impact on joint flexibility (78).

Sarcopenia, the definition of which was largely debated in the scientific community, is defined according to the European Working Group on Sarcopenia and Older People (EWGSOP) criteria as the presence of low muscle mass, plus low muscle strength, or low physical performance (79). The decline in muscle mass is believed to start between the ages of 30 and 40 with an estimated 10% muscle mass loss by the age of 50 (80). Thereafter, the loss is estimated to be around 1–2% per year. As for muscle strength, its rate of decline is about 3 times larger than that of muscle mass after the age of 50 (80). This age-related decline in skeletal muscle mass is attributed to an imbalance between muscle protein synthesis and breakdown rates, resulting in a negative muscle protein balance. Adequate dietary protein intake promote muscle protein synthesis rates. However, aging has been associated with a reduced muscle protein synthetic response to protein intake, termed “anabolic resistance” (81, 82). Physical activity performed before protein intake increases the use of protein-derived amino acids for postprandial muscle protein accretion in senescent muscles (83). Thus, the level of habitual physical activity is fundamental to maintain the anabolic responsiveness to protein intake with aging (84) and, ultimately, support healthy aging. There are other age-related confounding factors accelerating muscle aging, such as illness and accidents. The impact of these factors is well represented in the catabolic crisis model proposed by English et al. (85) which captures the deleterious effect of punctual episodes of illness or inactivity on the traditional sarcopenic model.

### 3.6. The cardiovascular system

Cardiovascular health is determined by the health state of the heart and the vasculature which regulate blood flow and blood pressure, ensuring adequate nutrition and oxygenation to all organ systems (86).

Vascular aging is a natural progressive change in the structure and function of the vasculature leading to a decline in arterial compliance and increased arterial stiffness or “hardening” and “thickening” of the arteries (87). Age is one of the greatest risk factors for cardiovascular disease which remains the leading cause of death in most countries; but environmental components (unhealthy diets, sedentary lifestyles, smoking, pollution, stress) may lead to premature deterioration of cardiovascular homeostasis and metabolic disturbances, ultimately leading to the early onset of disease in the vasculature and the myocardium (heart muscle).

The effect of aging on arterial stiffness and associated factors has been investigated in animal and human studies showing that, regardless of other cardiovascular risk factors, primary aging is an independent promotor of vascular aging (88). For instance, between ages of 20 and 90, arterial wall thickness increases two to three-fold (17, 89). Associated with these structural and functional changes in the vasculature is a significant age-related decline in endothelial function. Studies on healthy adults of different ages show a significant negative effect of aging on endothelium-dependent vasodilation, indicating endothelial dysfunction (90). The Framingham Heart study (91) demonstrates that aging is the strongest independent correlate of endothelium-dependent vasodilation. Other studies suggest that age-related endothelial changes throw the vascular system into a vicious circle where its effect is compounded by hypertension, inflammation, and lipid build-up, further increasing the risk of cardiovascular events (90). The direct result of impaired endothelial function is a decreased blood flow, also shown to decline with age. The rate of the decline is gender-specific. Males reach the onset faster than females, and after onset, loss of function is accelerated for females (92).

Another factor which compromises blood flow and circulation is the age-related reduction in cardiac output, mainly due to the effect of age on the number, function, and regeneration of cardiac cells. Indeed, the regenerative capacity of cardiac cells decreases from 1 to 0.4% between ages 20 and 75 (93). The loss of cardiac myocytes and an increase in fibrosis of the myocardium lead to a reduction in cardiac output and performance (93). This loss in cardiac output may stimulate the heart to produce muscle mass. Although this mechanism may provide an effective short-term solution, it is detrimental on the longer term and contributes to slow the propagation of the electric impulse. Like for vascular aging, gender-specific differences have been observed in the patterns of cardiac aging, which could be related to both hormone-dependent or -independent mechanisms (94).

Lipid build-up, mentioned above as a factor affecting vascular health, plays a role in plaque initiation and progression. The evolution of atherosclerosis starts with fatty streaks which form plaque along with fibrous elements, smooth muscle cells, and inflammatory cells such as T-lymphocytes (95). Aging was shown to affect these components putting older adults at an increased risk of developing severe atherosclerotic plaques (96). First, lipid levels, especially LDL-cholesterol (LDL-C), rise with age by up to 60% between ages 15–19 and 75–79, as reported in the Framingham Study (97). Second, arterial smooth muscle cells become increasingly disarrayed as do elastic fibers. Third, inflammation and oxidative stress, known to play an enhancing role in atherosclerosis, progressively escalate with age (87). Finally, the degree of plaque calcification was reported to be significantly higher in older versus younger adults (98), and the burden of calcified plaques (>50% of plaque tissue calcified) 16-fold higher in older than in younger adults (99).

As mentioned earlier, there is a closely related interplay between cardiovascular homeostasis and metabolic disturbances. Results from the Baltimore Study of aging highlight that there is an obvious age-dependent increase in glucose response and fasting blood glucose between ages 30 and 70. However, this increase is less striking after age 70. The authors argue that, in accordance with other studies, this worsening trend in glucose control is related to an increase in fat mass and a reduction in physical fitness, mainly due to unhealthy dietary patterns, sarcopenia, menopause, among other conditions typical of aging (100). Therefore, once again, the compounded effect of lifestyle-associated secondary aging, and primary aging, worsens insulin resistance and glucose control in the older adult.

## 4. Key strategies to promote healthy aging

Modifying the aging trajectory may be achieved by identifying a series of important players in the aging process and targeting them through interventions such as diet and lifestyle modifications which would help curb the functional decline in different biological systems. Drawing from the growing research on physiological changes associated with aging, we highlight selected targets and strategies for the maintenance of good health during aging.

### 4.1. Improving nutrient intake and metabolism

#### 4.1.1. Addressing inadequate intake

Older adults are prone to insufficient energy, macronutrient (namely protein), and micronutrient intakes, more significantly after the age of 65 years, often leading to nutrient deficiencies. The most common ones are deficiencies in vitamin B12, Iron, vitamin D and Calcium (14). Driven by intrinsic factors such as dental health, mental health, and digestive discomfort, or extrinsic factors such as social isolation and financial instability, diet quality is generally reduced with aging and so is the nutritional status of older individuals (14, 101). From a food intake point of view, it has been shown that feelings of hunger are reduced with aging (23, 24). Although confirmatory research is needed, the decrease in hunger is likely a result of an imbalance in gut peptide levels, more specifically an increase in plasma cholecystokinin concentrations, consistently shown to be higher in older versus younger adults (102–105). If food intake is reduced due to blunted hunger signals, it could further be decreased by the post-prandial gut discomfort experienced by older adults. Indeed, gastroesophageal reflux, bloating and constipation are quite prevalent amongst adults 60 years and older (106–108), with esophageal motility shown to start declining as early as age 40 (17, 109). Interventions to improve hunger in the elderly have shown to increase energy and nutrient intake and reduce age-associated weight loss. These interventions include serving smaller energy-enriched portions, favoring liquid versus solid textures (e.g., smoothies), or even improving the meal environment (110). Micronutrient supplementation as well have shown benefits in improving micronutrient status in older adults (111, 112). Therefore, improving/supporting digestive comfort, ensuring adequate nutrient intake at all stages of aging, and increasing hunger in the elderly are relevant strategies to target a healthy nutrient intake in order to avoid age-associated nutrient deficiencies.

#### 4.1.2. Supporting digestion and nutrient absorption

All systems rely on the gastrointestinal tract for their supply of nutrients which cannot be ensured if ingested nutrients are not adequately digested and absorbed. The declining absorption of calcium, vitamin D, and vitamin B12, compounded with their reduced intake described earlier makes them nutrients of concern for the older adult population typically at risk of osteoporotic fractures and cognitive decline. In fact adults 50 years and older were shown to have suboptimal intakes of vitamin D and calcium in epidemiological studies (113, 114) comparing intakes with international recommendations of calcium (950 mg/day) (115) and vitamin D (20μg/day) (116). Meanwhile, Vitamin B12 inadequacy (recommended intake: 2.4 μg/day) seems to be driven primarily by a declining digestion and absorption of the nutrient rather than its insufficient intake (117). Therefore, proposing strategies to improve digestion and absorption of these nutrients could prevent specific nutrient deficiencies with deleterious health impacts on aging individuals. For instance, maintaining a healthy gut microbial environment known to promote an anti-inflammatory state and consequently promote digestion and absorption of nutrients constitutes a relevant target to increase nutrient availability from the gut. Indeed, intestinal microbiota were reported to play a key role in macronutrient digestion and absorption (118, 119). Similarly, ensuring optimal gastric acidity and pepsin levels to enable efficient digestion and absorption of nutrients such as vitamin B12 (120) is another avenue to support digestion and absorption of this important micronutrient which declines with age.

#### 4.1.3. Optimizing energy metabolism

Energy substrate utilization is altered with age as insulin sensitivity decreases (100) and anabolic resistance increases (81). The implications on glucose metabolism are an impaired glucose response to a meal and compromised glucose utilization in multiple organs including the brain (60). Fat metabolism is equally affected by aging as fat deposition is redistributed from subcutaneous to visceral fat. This is partly due to declining levels of sex hormones and the decreased ability of adipocytes to buffer dietary lipids, channeling fat deposition to the muscle and liver (121). Visceral fat is subject to oxidation, promoting a state of low-grade inflammation, further impairing insulin sensitivity and increasing the risk of chronic diseases (121). Finally, age-related anabolic resistance, especially in the postprandial state leads to blunted postprandial muscle protein synthesis, which if sustained, results in lower muscle mass and strength (84, 122). Although age-related reductions in protein digestion and absorption were not consistently shown (14, 123, 124), the reduction in intake and anabolic resistance to dietary protein associated with aging make protein metabolism an area of concern for older adults.

On a cellular level, mitochondrial energy metabolism is typically less efficient in senescent cells (125). Maintaining mitochondrial health and delaying dysfunction has been shown to promote energy homeostasis and may therefore prevent tissue damage and delay cellular aging.

In summary, energy metabolism occupies a prime position in the strategies designed to curb the aging trajectory and improve lifespan and health span.

### 4.2. Limiting inflammation

Low-grade inflammation is a ubiquitous condition in older adults related to multiple age-associated factors, namely oxidative stress, DNA damage, infection history and dysbiosis (35). Inflammaging is a main contributing factor to tissue damage and the decline in immunity, mobility, brain, heart, and gut health. Indeed, a pro-inflammatory state heightens the body’s vulnerability to pathogenic invasion, exposing it to the risk of disease. Higher blood levels of pro-inflammatory cytokines have also been linked to an increased loss of muscle strength, potentially through the degradation of myofibrillar proteins (75). Similarly, systemic inflammation is believed to activate innate immunity in the central nervous system (CNS) which may lead to neuroinflammation, a known factor in neurodegenerative disease (126). From a cardiovascular point of view, inflammation is involved in various stages of atherosclerosis and research shows that inflammatory markers such as CRP are good predictors of cardiovascular events regardless of blood LDL-cholesterol levels (127, 128) Finally, inflammation at the level of the gut promotes intestinal barrier permeability, weakening barrier function and consequently gut health. As such, inflammaging is a warranted target for the maintenance of good health and the delay of age-associated functional decline.

### 4.3. Mitigating oxidative stress

One of the most popular theories of aging is the free radical theory which stipulates that aging occurs as a result of the accumulation of reactive oxygen species (ROS) damage, leading to cellular dysfunction. Indeed, an elevation in cellular ROS levels coupled with a reduction in antioxidant capacity has been associated with aging (129). A physiological production of ROS is a core part of natural defenses, be it against invading pathogens (130), or as a protective mechanism through which antioxidant capacity is upregulated, for instance in the brain (126). However, an imbalanced redox status characterized by excessive ROS production and an accumulation of oxidative products leads to tissue damage and functional decline. In fact, high ROS production in the gut is associated with increased inflammation, low SCFA production, and dysbiosis, all deleterious to gut and systemic immunity (35). Oxidative stress is also a major contributor to the loss of bone mass and strength (14). In a review by Domazetovic et al., the authors explain that an overproduction of ROS, with increased oxidative stress as a result, induces osteoblast apoptosis which in turn activates osteoclast generation. This tendency toward bone catabolism is manifested as decreased bone strength (131), thus weakening the skeletal system. In the joint, an increase in ROS due to mitochondrial dysfunction leads to chondrocyte inflammation, apoptosis, matrix catabolism and calcification (132), affecting joint flexibility. In the brain, a high ROS producer, a decrease in antioxidant capacity means a loss of protection against high oxidative stress, exposing neurons to tissue damage and inflammation and leading to cognitive decline (126). Finally, oxidative stress plays an important role in the process of atherosclerosis, as oxidized lipids in the endothelium lead to the generation of ROS which contribute to atherosclerotic plaque and nitric oxide inactivation, hence reducing its bioavailability and beneficial effect on the endothelium. Equally to inflammation, management of oxidative stress is a relevant target to the different systems of the aging human body and therefore should be the focus of interventions.

### 4.4. Promoting gut microbiota balance

Gut microbial health is determined by its billions of residents acting locally as the first line of defense against invading pathogens and influencing the health of other biological systems through the gut-organ axes (16, 30, 37, 38). A healthy microbiota, generally characterized by high counts of Bifidobacterium and Lactobacillus species, compete with pathogens for adhesion to the mucosa and promote the development of immune cells. On the other hand, pathogens and pro-inflammatory cytokines can disrupt the stability of tight junctions and lead to increased gut permeability. It is this interplay between commensals and pathogens that limits infection and disease (38). With recent research shedding light on the communication between different organs and host immunity (16, 30), it is increasingly clear that immune dysfunction at the level of the gut may have consequences on the immune health of other organs (e.g., lung). This is mediated by the crosstalk between different systems through the so-called gut-organ axes, such as the gut-liver axis, gut-lung axis and the gut-brain axis. More specifically, immune systems in the gut and other organs communicate via the gut microbiota and its metabolites such as SCFA acting as signaling molecules (41, 133–135). Improving microbiota composition or the balance between beneficial microbiota and pathobionts thus represents a central target for the maintenance of gut barrier integrity, immunity, and subsequently, general health and wellbeing.

## 5. Factors modulating the path of aging

The debate around whether humans are programmed to live a certain number of years is an ongoing one with data from human studies proposing genetic makeup as a main player in aging and longevity (136). Studies in monozygotic and dizygotic twins have led to the hypothesis that genetic factors can explain about 25% of the variation in human longevity (137–139). This was supported by sibling and extended family studies which concluded that genetic factors were directly linked to lifespan, after adjusting for family environment (140, 141). Moreover, specific mutations in genes associated with DNA repair, telomere conservation and free radical control were found to have a modulatory effect on longevity (136). Fewer studies exist on the relationship between lifestyle and longevity *per se*. Findings reveal negative correlations between a healthy lifestyle (combined diet and physical activity) and age-associated decline or all-cause mortality (142–145), as well as a positive correlation with longevity itself (143, 146). In that regard, the Healthy Aging Longitudinal study of Europe cohort (142), aiming to identify lifestyle patterns which could influence longevity, followed 70- to 90-year-old individuals for 10 years and collected data pertaining to diet and lifestyle, as well as disease, disability, and mortality. The authors reported a strong inverse relationship between a healthy lifestyle pattern and all-cause mortality, that pattern being a combination of adhering to the Mediterranean diet, being physically active, consuming alcohol in moderation and not smoking (142). Another observational study investigating longevity of the US population as a function of behavioral factors reported a significantly higher life expectancy in individuals with healthy behavioral profiles versus the total population (143). Briefly, a healthy behavioral profile characterized by the absence of obesity, smoking and heavy drinking increased life expectancy by 7 years and delayed the onset of disability by 6 years as compared to the general US population.

While research on promotors of longevity is rather limited, there is a larger body of evidence on factors enhancing different aspects of health in aging. These factors can be categorized into unmodifiable or fixed such as gender and genetics, and modifiable such as diet and physical activity.

### 5.1. Unmodifiable factors

#### 5.1.1. Gender

Gender differences in aging are particularly observed in the area of mobility whereby peak bone mass is lower and the decline in bone density steeper in women than in men. However, gender becomes a differentiating factor in multiple biological systems after menopause. First, hormonal shifts affect nutrient utilization, altering fat metabolism predisposing women to abdominal obesity and increasing their risk for cardiovascular disease (147). Some micronutrients such as vitamin C and calcium are also affected by the loss of the protective effect of estrogen during menopause. The utilization of vitamin C increases to counterbalance the increase in oxidative stress typical of menopause (148). Similarly, as bone resorption increases, calcium needs are increased to prevent osteoporotic fractures. Second, from a digestive health perspective, menopausal changes have been shown to alter gut microbiota, causing dysbiosis with an increase in the Firmicutes to Bacteroidetes ratio (149). Finally, the menopausal transition puts women at a higher risk of depression, the effect on mood being partly explained by the loss of the beneficial effect of estrogen on serotonin and other mood regulating hormones (150).

Therefore, around age 50, gender differences become increasingly striking and must be taken into consideration when designing interventions for healthy aging.

#### 5.1.2. Genes

Genome-wide association studies have greatly increased knowledge around genetic variations and their modulation of chronic disease risk. Genes associated with physiological and metabolic pathways may therefore predict the path to age-related disorders (136). For instance, numerous gene variants were identified to be associated with a higher risk of cardiovascular disease (151), and its five main risk factors, diabetes, obesity, dyslipidemia, smoking (nicotine dependence), and hypertension (152). Equally, numerous single nucleotide polymorphisms were associated with bone mineral density, osteoporosis and osteoporotic fractures (153). Finally, cognitive decline has been the focus of recent investigations identifying genetic variations associated with Alzheimer’s disease (154, 155). Considering the role that genes play in determining longevity and their association with the risk of age-related disease, it is safe to say that genetics contribute significantly to determining both lifespan and health span.

### 5.2. Modifiable factors

In their review on human longevity, Passarino et al. argue that “it takes two to tango,” genetics and lifestyle going hand in hand to improve or worsen the trajectory and outcomes of aging (136). Studies have shown that genetics can account for 20–30% (136, 139, 156) of longevity, leaving 70–80% to be modulated by the environment. Indeed, diet, lifestyle, and the environment appear to be driving longevity in regions of Japan (Okinawa) (157), Greece (Icaria), Italy (Ogliastra) and Costa Rica (Nicoya Peninsula) (158), identified as “blue zones,” the world’s longest-lived cultures (159). Therefore, regardless of the competitive advantage hidden in our genes, lifestyle is most definitely linked to the way we age.

#### 5.2.1. Nutrition

Nutritional modifications have repeatedly shown to significantly impact the risk of various age-related diseases, and nutritional solutions may be designed to target the hallmarks of aging. Although personalized nutrition is increasingly considered as the optimal means to promote healthy aging, research on its long-term impact on disease prevention remains limited (160). On the other hand, some dietary solutions discussed below are well-supported in terms of their role in the prevention of age-related diseases and the promotion of healthy aging in the adult population.

##### 5.2.1.1. Dietary patterns

Aging is characterized by a declining nutritional status due to reduced intakes of nutrient-dense foods, lower intestinal absorption, and impaired nutrient metabolism. Nutrient requirements of older adults (60 years and over) and dietary guidelines have therefore been adapted to accommodate for these differences with young individuals (161). That said, dietary changes should start prior to the first signs of aging since the onset of decline in different systems can start as early as age 40. More importantly, these requirements are often unmet due to the physical, social, and environmental difficulties that older adults face, more particularly the late elderly (over 75 years). Promoting a balanced diet and nutritional solutions to meet requirements is therefore important. For instance, meeting protein needs could be facilitated by offering solutions that provide high-quality protein and protein dense products adapted to age-related conditions (e.g., blunted feelings of hunger). Similarly, micronutrient supplementation and fortification could be considered to counterbalance inadequate intake linked to an unbalanced diet.

Unbalanced or unhealthy diets such as the western diet typically contain high levels of salt, saturated fats, refined sugars and are generally poor sources of dietary fiber and micronutrients. Globally and over the past decades, diets have evolved from traditional healthy diets to westernized diets which have shown deleterious effects on health (162). More specifically, westernized diets negatively impact the composition of gut microbiota, powerful metabolic regulators, whose functions have repercussions on all body systems, and general health (162, 163). As mentioned earlier, gut microbiota composition and function are altered with age which further emphasizes the importance of a healthy, microbiome-promoting diet in this population.

Healthy dietary patterns, which can be defined by a variety of healthy eating indices, are generally rich sources of fruits, vegetables, legumes, whole grains, nuts, low-fat dairy and fish, and provide a healthy fat profile characterized by low saturated and high unsaturated fats, including polyunsaturated omega-3 fatty acids (n-3 PUFAs) omega-3s. One such pattern is the Mediterranean diet which has particularly been explored in studies investigating the impact of nutrition on health markers in older adults. Findings from these studies associate the Mediterranean diet with a favorable inflammatory profile (164), lower risk of osteoporotic fractures (165), higher muscle mass (166) and better mobility performance (167). The Mediterranean diet was also linked to a lower prevalence of cognitive decline, dementia and Alzheimer’s disease (168). Importantly, a cause-and-effect relationship between the Mediterranean diet and cardiovascular health was established in a randomized clinical trial demonstrating a lower rate of cardiovascular events following a plant-based Mediterranean-type intervention diet versus a western diet (169). Other dietary patterns having shown beneficial effects on cardiovascular health are the DASH (Dietary Approach to Stop Hypertension) diet, and the Portfolio diet. The DASH diet emphasizes fruits, vegetables, whole grains, and low-fat dairy products. It also limits the intake of sugar-sweetened foods and beverages, red meat and added fats. Its demonstrated efficacy on blood pressure has been reported in numerous clinical trials (170). The portfolio diet is a plant-based dietary pattern promoting the intake of nuts, viscous fiber and vegetable protein and supplemented with plant sterols. The portfolio diet is known for its reducing effect on LDL-cholesterol and was ranked first amongst efficacious dietary patterns for secondary prevention of cardiovascular disease (subjects with pre-existing CVD) in a report of the National Heart foundation of Australia in 2017 (171). Finally, the MIND diet, a pattern that is increasingly popular amongst scientists and practitioners, is a combination of the Mediterranean and the DASH diets targeting the driving processes behind cognitive decline (172). Although dietary patterns mentioned here are supported by variable levels of scientific data, they share common characteristics combining healthy energy substrates (whole grain carbohydrates, n-3 PUFAs, plant-based protein), reducing trans-fats, saturated fats, refined grains and added sugars, while providing good sources of antioxidants. Components of these diets are natural bioactives with proven beneficial effects on the age-associated decline of different systems. Here, we highlight a few of these components.

##### 5.2.1.2. Micronutrient supplementation

Meeting micronutrient needs through a healthy diet ensures a good functioning of all body systems. Vitamins A, C, D as well as copper, iron, selenium and zinc are supported by sufficient evidence regarding their role in maintaining immune function (173). In addition, clinical trials on the supplementation of zinc, vitamin C, vitamin E as well as multiple micronutrients in the aging population improve immune activity, reduce the incidence and morbidity of respiratory tract infections, and may improve the response to vaccination (29). In addition to their role in immunity, B vitamins, specifically B6, B9 (folate) and B12 are central to maintaining cognitive function as deficiencies are linked to cognitive impairments (174, 175) and supplementation studies have shown improvements in global cognition (176). Other micronutrients such as calcium and vitamin D, magnesium, and potassium, highly available in the Mediterranean and the DASH diet, are efficacious for bone strength, colonic motility, and blood pressure, respectively.

Therefore, filling the micronutrient gap is essential for the maintenance of immune and brain health, especially in the context of deficiencies associated with older age. However, proper individual assessment should be made before supplementation as some elderly people may already consume in excess micronutrients such as vitamin A or folate (45).

##### 5.2.1.3. n-3-PUFAs

The evolution of heart healthy diets was such that dietary fats were first demonized before regaining their place in primary prevention, with great emphasis on quality rather than quantity. Polyunsaturated fatty acids, and especially n-3 PUFAS have since then been extensively researched and shown beneficial across different aspects of health. Marine n-3 PUFAs eicosapentanoic acid (EPA) and docosahexanoic acid (DHA) (177, 178), found in the Mediterranean diet through an adequate intake of fish, have consistently shown an inverse relationship with plasma pro-inflammatory markers such as IL-6, CRP, and TNF-a and a positive association with anti-inflammatory markers such as IL-10 and TGF-b in young and older adults. Their mechanism of action is mainly through displacing arachidonic acid, the main substrate for the production of eicosanoids, thus reducing the production of pro-inflammatory cytokines (179). In light of the recent COVID-19 pandemic affecting older adults in particular, Calder et al. suggest the inclusion of EPA and DHA in the treatment of affected patients to avoid the cytokine storm (30). In fact, pre-clinical models of lung injury support the role of EPA and DHA in resolving inflammation and a recent meta-analysis of clinical trials concluded that n-3 PUFAs lead to the reduction of mechanical ventilation and ICU stay (180, 181).

The targeted effect of n-3 PUFAs on inflammatory pathways emphasizes the relevance of these fatty acids in the prevention of low-grade inflammation and its deleterious effect on immune, brain, musculoskeletal, digestive and heart health.

##### 5.2.1.4. Polyphenols

Polyphenols are phenolic compounds which are ubiquitous in the plant kingdom. Polyphenols from commonly consumed foods in healthy diets (e.g., in olive oil, berries, red wine) may contribute to reduce the oxidative burden of aging cells, promoting autophagy (182) and thus delaying cell senescence. Autophagy is a cellular housekeeping process that rids the cell of old and damaged organelles, improving cellular functioning and mitigating cellular oxidative stress. Cocoa and olive polyphenols are effective at reducing oxidative stress and inflammatory markers in young and older adults (35), resulting in significant improvement in blood lipids (olive oil polyphenols) and blood flow (cocoa polyphenols). Meanwhile, curcumin (from the turmeric root) (183) demonstrated a protective effect on the cartilage manifested through reduced joint pain and increased functionality in older adults, explained by reduced levels of inflammation and oxidative stress. Given the pivotal role of oxidative status and autophagy in cellular aging (129), polyphenols should be part of the prevention strategy for healthy aging.

##### 5.2.1.5. Probiotics and prebiotics

Microbiota-targeting solutions have been extensively investigated in recent years for their local (digestive) and systemic effects. The latter include improvements in immune, cardiovascular, skeletal, and cognitive health.

Probiotics are defined as live microorganisms that, when administered in adequate amounts, confer a health benefit on the host (184). Prebiotics are substrates that are selectively utilized by host microorganisms conferring a health benefit through altering their composition or function specifically (185). Probiotics are established key players of immune health as discussed in the recent review by Bosco and Noti (31). The authors evaluated findings from 31 studies on probiotic interventions in elderly subjects reporting that two thirds show a positive, albeit strain-specific effect on the immune system. Benefits included improving response to vaccination and protecting against bacterial infections (31). Probiotics are first and foremost the guardians of the intestinal barrier, protecting its integrity and forming a first line of defense against invading pathogens. However, they also have an important role to play locally on digestion and absorption. For example, L. delbrueckii subsp. bulgaricus and S. thermophilus in fermented milk were shown to improve lactose digestion, a claim that was accepted by the EFSA in 2010 (186). Probiotics have been repeatedly suggested to impart cardiometabolic benefits such as lipid and glucose-lowering (187, 188) by modulating gut microbiota and SCFA production, with a purported effect on oxidative stress and low-grade inflammation (189). From a bone health perspective, a daily ingestion of L. reuteri attenuated bone loss in older women with low bone mineral density (190). Finally, psychobiotics, a relatively new class of probiotics that confer a mental health benefit to the host (191), have already shown promising effects on cognition and mental state, acting via the gut-brain axis by modulating neurotransmission, neurogenesis, and neuroinflammation (192). Probiotics were shown to decrease depressive symptoms in individuals with depression (193), increase neurocognitive performance in healthy adults age 18 to 40 (B. longum) (194), reduce anxiety symptoms in adults age 18 to 65 (L. casei Shirota) (195), and improve cognitive performance in older adults (L. Helveticus) (195), agreeing with findings from younger populations.

Notwithstanding the large body of evidence supporting the efficacy of probiotics on immune, digestive, cardiovascular and brain health, there is a need for additional targeted clinical trials to establish strain-specific effects on each of these benefits.

Just like probiotics, prebiotics are microbiota-related solutions having demonstrated multiple benefits for human health. Complementing their well-established role in balancing the intestinal microbial ecosystem and consequently general health, specific effects of inulin-type of fructans and galacto-oligosaccharides (GOS) were reported on decreasing serum pro-inflammatory markers (196) and increasing immune cell activity (197). Prebiotics have also been investigated in mobility showing promising avenues for bone health. Briefly, fructooligosaccharides (FOS), inulin, and GOS increase calcium absorption in postmenopausal women (198–201) while FOS and inulin further reduce bone resorption markers (202) and soluble corn fiber increase bone formation markers (203) in the same population. However, there is no evidence to date that this translates into increasing bone mineral density (BMD) or decreasing the risk of fracture. This is possibly due to the short-term nature of before mentioned studies which raises the need for longer-term research investigating the impact of prebiotics on bone strength and mobility.

#### 5.2.2. Physical activity

Physical activity which includes exercise, in general terms, is commonly known for its health benefits and is an obvious component of strategies aiming to improve the general health of aging adults. In fact, according to the WHO, older adults should perform 150 min of moderate-intensity exercise or at least 75 min of vigorous exercise per week (204). The most obvious impact of exercise is on mobility where regular training can improve muscle structure and function in older adults to match that of men four decades younger (205). In addition, exercise increases the number of muscle satellite cells (206), attenuates insulin resistance (207), improves mitochondrial capacity (208), and allows for greater use of dietary protein–derived amino acids for *de novo* muscle protein accretion in senescent muscle (83). In bones, it increases mechanical stress and physical loading which in turn increases bone mass and density through activating bone formation and reducing resorption (209). With regards to immunity, moderate to vigorous activity enhances immunosurveillance by recirculating immunoglobulins, immune cells, and anti-inflammatory cytokines (210, 211). On the other hand, exercise immunology studies show that the impact of exercise on immune function follows a J-shaped curve where repeated moderate exercise enhances immune health, decreasing incidence of illness and dampening inflammation, while prolonged heavy exertion leads to an increased risk of illness. This is due to a transient state of immune dysfunction, inflammation, and oxidative stress which can last up to several days during recovery (210). The same pattern is observed in digestive health whereby regular moderate exercise has positive effects on gut motility and microbiota composition whereas exertive exercise can have deleterious effects on gut health (212). In terms of the decline in cognitive and cardiovascular function, preventive effects of regular moderate exercise share common pathways, namely an increase in blood flow and a decrease in inflammation and oxidative stress. In fact, cardiovascular training is considered as one of the most effective strategies to prevent cognitive decline (54). In a meta-analysis of exercise interventions in adults 18 to 90 years of age, Lin et al. report a beneficial effect of exercise on cardiorespiratory fitness (CRF) and markers of cardiovascular disease (213). The latter included blood lipids, inflammatory markers, insulin resistance and hemostatic factors involved in endothelial function and blood pressure. On the brain health front, studies have demonstrated that physical activity increases cognitive performance, more specifically in verbal memory and attention. Moreover, amongst aerobic exercises recommended for older adults, dancing has shown a superior effect, possibly differentiating itself by the additional positive emotional impact of music and the continuous engagement in cognitive and motor learning (214).

#### 5.2.3. Sleep quality

Aging is accompanied by disturbances in the circadian rhythm which lead to sleep disturbances (215). Nocturnal sleep is a necessary physiological process which plays an important role in physical and mental recovery (215). From a brain perspective, the restorative role of sleep involves brain tissue restoration, metabolite clearance, and memory consolidation (216). Sleep disturbances are common in older adults (40–50% of adults over 60) and seem to go hand in hand with cognitive and mood disorders (216, 217). In fact, sleep loss has consistently shown to impair cognitive performance, namely attention and executive control while sleep has proven to be a process that promotes memory stabilization and integration (216) in the general population. How these functions are affected in the context of the aging brain was addressed in the review by Scullin et al. suggesting that good sleep quality can promote cognitive function in young and middle-aged adults and protect against age-related cognitive declines (216).

The relevance of sleep and processes that occur during the sleeping state is often seen as exclusive to brain health. However, the impact of sleep quality is ubiquitous to all systems of the human body although significant gaps in knowledge remain as to the clear impact of sleep quality on different aspects of health, namely immune, digestive, musculoskeletal, and cardiovascular health. The relationship between sleep and immunity appears to be bidirectional whereby sleep restriction increases markers of inflammation such as IL-6, IL-1b, and TNF-α, while at the same time, increased levels of inflammatory markers result in disturbed sleep (218, 219). Although not consistent in the literature, the causal relationship between sleep quality and immune function is supported by pre-clinical studies showing deleterious effects of sleep loss on inflammation, and clinical studies revealing an impact of sleep quality on adaptive immune responses, more specifically in the context of vaccination, whereby sleep deprivation was seen to attenuate antibody responses (220). Equally bidirectional is the relationship between sleep and digestive health. It is now well-recognized that digestive perturbances and diseases such as inflammatory bowel disease and Crohn’s disease are associated with fatigue (221, 222). Furthermore, recent work has shown that sleep quality is associated with gut dysbiosis, and that sleep efficiency is positively associated with microbiota diversity (223). This association is suggested to be mediated by the HPA (hypothalamus pituitary adrenal)-axis (224).

Finally, circadian dysregulations caused by sleep deficit modulate circadian hormones involved in nutrient metabolism and consequently, cardiometabolic health (225, 226). St-Onge et al. examined the strength of the evidence behind the role of sleep in cardiometabolic health in a review demonstrating that epidemiological and clinical trials support a deleterious effect of sleep restriction on insulin resistance, blood pressure, inflammatory markers, and cardiovascular risk in general (227). Overall, the available clinical trials suggest that sleep deprivation can be deleterious to cardiovascular risk factors such as blood pressure (228) and endothelial function (229).

#### 5.2.4. Physical and mental stress

Stress can be defined as the way the body responds to a challenge, be it physical or mental. It is also defined as a state of disharmony caused by these challenges, defined as “stressors” (230). Whether it is performance at work or in athletic competitions, a significant life change or a traumatic event, the body’s response to stress includes an increase in heart rate and blood pressure, stimulation of stress hormones and other stress-related pathways (230). Although stress represents an asset from an evolution point of view, rooted in the fight or flight response, chronic stress over the lifespan has proven to be deleterious to health. Indeed, epidemiological data shows that individuals with chronic stress have signs of decreased immune performance manifested in poorer vaccination responses, impaired wound healing and weaker control of latent viruses (231). This relationship was also observed in the experimental setting where a higher risk of developing a cold was associated with the occurrence of a recent life stressor in men and women exposed to the rhinovirus (232). In the context of aging, the accumulation of stressors throughout life and the resulting chronic stress observed in aging adults is believed to greatly contribute to the weakening of the immune system and the development of chronic diseases (233). To test the impact of life stressors on immunosenescence, Puterman et al. followed healthy women aged 50–65 years over one year and measured leukocyte telomere length as a marker of aging immune cells. The authors report that for every life stressor in that year there was an incremental decrease in telomere length, and that this decrease was moderated by other modifiable risk factors such as diet, physical activity and sleep (234). Other aspects of health such as digestive and brain health are equally affected by chronic stress. Stress can impact the colonic motility and gut microbiota composition, reducing Lactobacilli counts and increasing adhesion of pathogenic bacteria (235). In chronic gastrointestinal conditions such as inflammatory bowel disease (IBD), evidence shows that stress alters intestinal mucosa permeability and increases inflammation, worsening outcomes of the disease (236). Chronic stress has been strongly correlated with cardiovascular disease. The INTERHEART study has shown that psychosocial factors were significantly related to acute myocardial infarction to the same extent as the more traditional risk factors (237). Finally, an obvious effect of stress is on affect and mood and using cognitive-behavioral stress management has shown the best evidence in reducing physiological stress, as measured by plasma cortisol levels (238). Overall, research to date emphasizes the importance of managing physical and psychological stress to alleviate the burden on immune, digestive, and mental health. Aging adults have higher exposure to the deleterious effects of stress due to the accumulation of lifelong stressors and therefore stress management is particularly important in this population.

## 6. Summary and conclusion

There is a general awareness of the rapidly aging global population and its impact on health, social and environmental systems. Advances in medicine and science have enabled us to increase the average human lifespan by providing treatments for the most fatal diseases of old age such as cancer and heart disease. Nevertheless, living long is not always equal to living well. Hence, to reduce the burden of old age, the focus should be placed on the health span rather than the lifespan. This is only attainable through optimal development and early prevention before the surge of “calls to action” or visible signs of impairment. The World Health Organization defines healthy aging as the “process of developing and maintaining the functional ability that enables wellbeing in old age.” Understanding the decline in functional ability of each biological system and identifying common biological targets and strategies based on the hallmarks of aging are key to delaying age-associated decline. These targets have been shown to be involved in the aging process and to be modulated by dietary and lifestyle changes. Therefore, improving dietary patterns, promoting regular moderate physical activity, improving sleep quality, and reducing life stressors are likely to modulate the path of aging through their action on aging targets, namely energy metabolism, microbiota function, inflammation, and oxidative stress. Finally, a holistic approach combining nutrition and lifestyle modifications is the optimal way to make an impact on the health span of older adults and thus improve their quality of life. The holistic approach should take into consideration the interplay between different factors such as socio-economic status, health and social service systems, physical and social environment, cultural, personal and behavioral determinants.

## Author contributions

AK and LD-C performed the writing – original draft preparation. M-CF, M-NH, AH, PD, GB, NP, DZ, NB, and KV provided the critical scientific and intellectual inputs and critically revised the manuscript. All authors have read and agreed to the published version of the manuscript.
